# Clinical Potential of Extracellular Vesicles in Type 2 Diabetes

**DOI:** 10.3389/fendo.2020.596811

**Published:** 2021-01-21

**Authors:** Jie Liu, Xin Sun, Fu-Liang Zhang, Hang Jin, Xiu-Li Yan, Shuo Huang, Zhen-Ni Guo, Yi Yang

**Affiliations:** ^1^ Stroke Center & Clinical Trial and Research Center for Stroke, Department of Neurology, The First Hospital of Jilin University, Changchun, China; ^2^ China National Comprehensive Stroke Center, Changchun, China; ^3^ Jilin Provincial Key Laboratory of Cerebrovascular Disease, Changchun, China

**Keywords:** type 2 diabetes, extracellular vesicles, mechanism, diagnosis, treatment

## Abstract

Type 2 diabetes (T2D) is a major public health disease which is increased in incidence and prevalence throughout the whole world. Insulin resistance (IR) in peripheral tissues and insufficient pancreatic β-cell mass and function have been recognized as primary mechanisms in the pathogenesis of T2D, while recently, systemic chronic inflammation resulting from obesity and a sedentary lifestyle has also gained considerable attention in T2D progression. Nowadays, accumulating evidence has revealed extracellular vesicles (EVs) as critical mediators promoting the pathogenesis of T2D. They can also be used in the diagnosis and treatment of T2D and its complications. In this review, we briefly introduce the basic concepts of EVs and their potential roles in the pathogenesis of T2D. Then, we discuss their diagnostic and therapeutic potentials in T2D and its complications, hoping to open new prospects for the management of T2D.

## Introduction

In 2019, there were approximately 463 million adults with diabetes worldwide, among whom 90%–95% had type 2 diabetes (T2D) (1). T2D is characterized by peripheral insulin resistance (IR) and insufficient pancreatic β-cell mass and function (2–4). These disorders then disrupt systemic metabolic homeostasis, placing an enormous burden on diabetic patients and the healthcare system.

**Figure 1:**
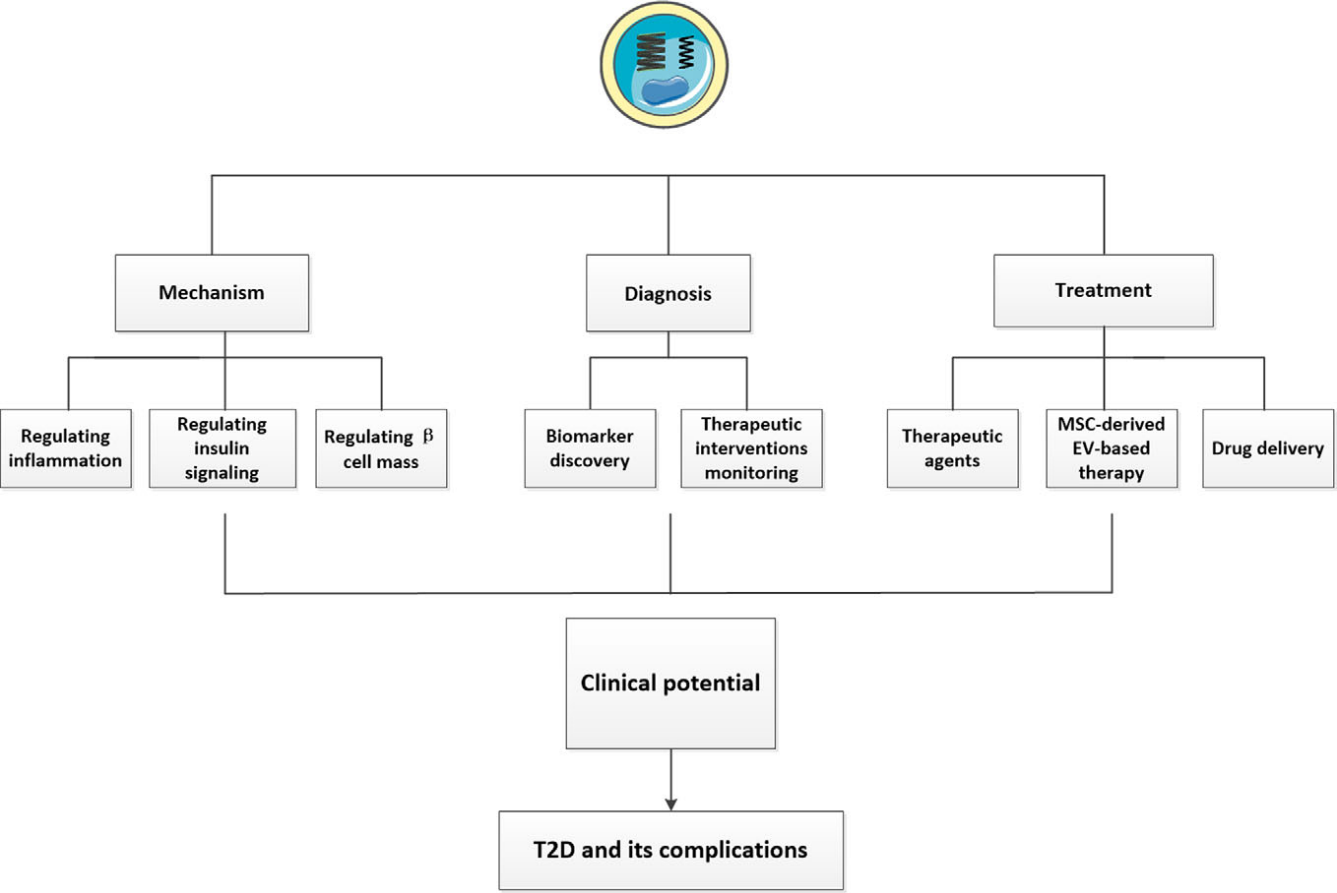
The clinical potentials of EVs in T2D and its complications. We showed the clinical potentials of EVs described in our review. First, EVs can influence the pathogenesis of T2D by regulating inflammation, insulin signaling and β cell mass. Second, EVs isolated from various body fluids may have huge potentials to be novel biomarkers of T2D and its complications. Some exosomal cargos altered following different treatments may open new prospects for monitoring the efficacy of therapeutic interventions and favor machinery discovery. The therapeutic potentials of EVs have also been presented. Using EVs or their mimics as suitable drug delivery system and MSC-derived EV-based therapy have been exploited a lot, in addition, some EVs possessed beneficial effects can also serve as potential therapeutic agents for T2D and its complications.

The specific mechanisms underlying the pathogenesis of T2D are complex and largely unknown. Current clinical therapeutic interventions for T2D mainly rely on hypoglycemic drugs, insulin supplementation, or other symptomatic treatments, which are invalid in treating the root of the disease. Thus, a deeper understanding of the pathological process of T2D might provide new ideas for treating T2D by modulating the disturbed ways, but not only alleviating symptoms. Extracellular vesicles (EVs) are small vesicles released by nearly all cell types. They can deliver various kinds of cargos such as proteins and nucleic acids to nearby or distant recipient cells (5, 6), thus mediating a new cell-to-cell communication (7). Recently, it was discovered that a number of abnormal EVs can play important roles in the pathogenesis of T2D. In 2018, Freeman et al. observed altered levels of insulin signaling proteins in EVs and increased secretion of EVs from patients with severe IR and β-cell dysfunction (8). A consistent trend was also reported in which the levels of circulating EVs were positively related to homeostasis model assessment β-cell function (HOMA-β) (9). Low grade chronic inflammation has been gradually recognized as a universal mechanism in the pathogenesis of T2D (10). Thanks to their ability to carry pro-inflammatory molecules, EVs can also act on different tissues to induce systemic inflammation. Considering their important functions, increasing attentions have focused on EVs as attractive diagnostic and therapeutic tools for T2D and its complications (**Figure 1**). In this review, we summarized the current advances concerning the roles of EVs in T2D and discuss their prospects to be used in the management of T2D and its complications.

## Biogenesis of EVs

The term “EVs” describes lipid bilayer enclosed structures typically ranging in size from ~30 to 400 nm that contain various biomolecules, such as proteins, lipids, RNAs, and DNA. These small vesicles can be released by almost all cell types (11) in response to various types of stimulation, and their altered expressions have been showed to play essential roles in regulating a number of biological processes such as angiogenesis, inflammation, immune responses, and so on (12, 13).

**Figure 2:**
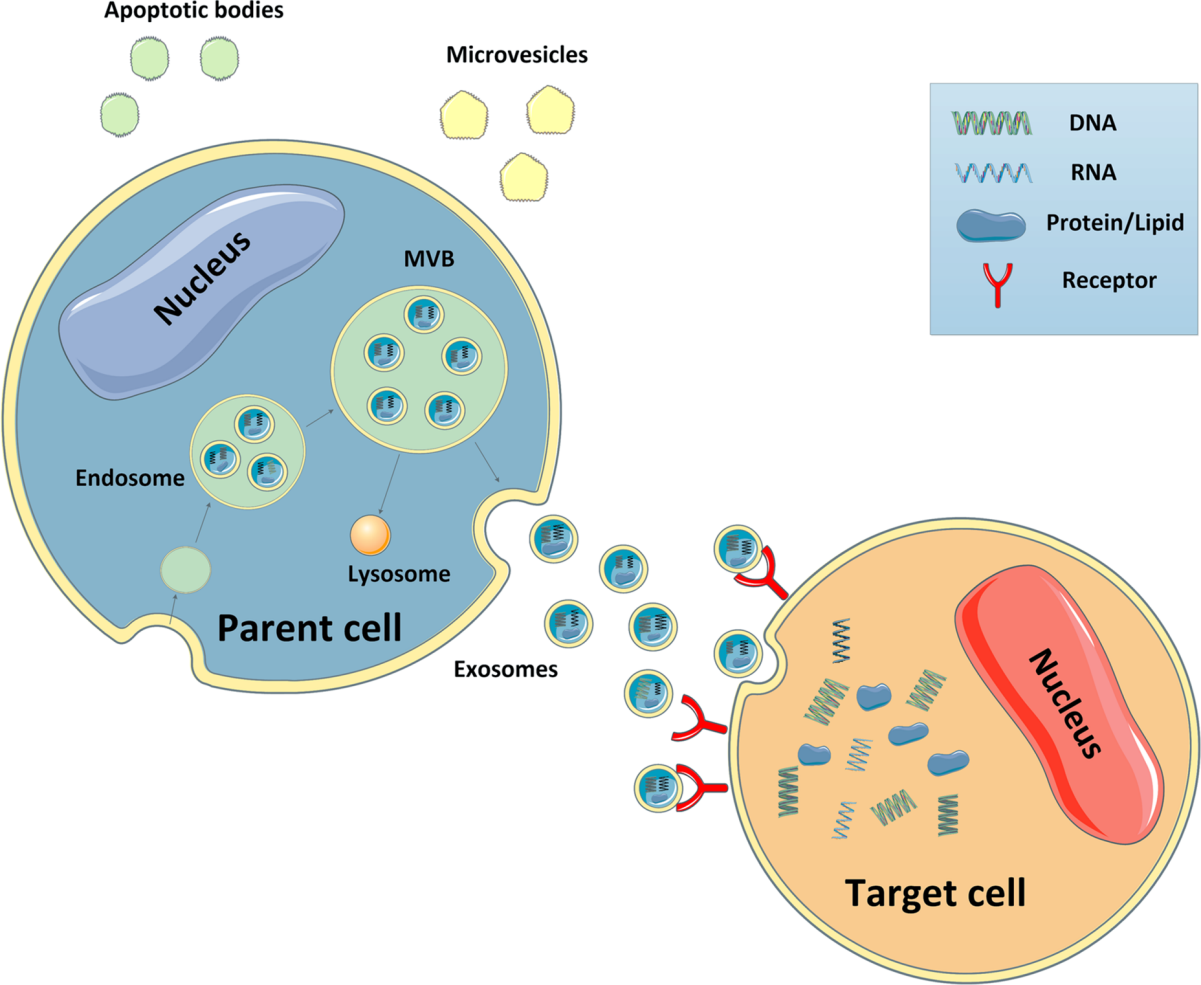
Biogenesis of EVs and target cell interactions. Microvesicles are generated by plasma membrane budding. Apoptotic bodies are formed by outward budding of the plasma membrane of apoptotic cells. Exosomes are generated within the cells through the endosomal pathway. The cellular contents form intraluminal vesicles which aggregate to form larger vesicles known as MVBs. Some of these MVBs then fuse with lysosomes to degrade cellular components, whereas others fuse with the membrane and are secreted into the extracellular milieu. Subsequently, these exosomes travel to neighboring and distant organs and impact the cellular function of recipient cells.

According to their biogenesis, EVs can be classified into three distinct subtypes, apoptotic bodies, microvesicles (MVs), and exosomes (14). The traditional strategy used to isolate each type of EV is differential ultracentrifugation. In recent years, new approaches have been established, such as density-gradient ultracentrifugation, which enables the separation of more specific EV populations. According to current views, MVs are defined as EVs with a diameter of 200–2,000 nm generated by plasma membrane evaginations. They were originally described as subcellular material derived from serum/plasma platelets (15). Apoptotic bodies, with a diameter of 500–2000 nm, are formed by outward budding of the plasma membrane of apoptotic cells, and some nucleic materials and proteins from apoptotic cells can also be included (16). Unlike other EVs, exosomes, with a diameter of 30–100 nm, are generated within cells through the endosomal pathway. First, the cellular contents form intraluminal vesicles (ILVs) which aggregate to form larger vesicles known as multivesicular bodies (MVBs). The mechanisms mediating this process can be divided into two distinct pathways: the endosomal sorting complex required for transport (ESCRT)-dependent pathway (17), and the ESCRT-independent pathway (18). The ESCRT-dependent pathway requires the formation of a complex by ESCRT, the sorting protein, Vps4, and the constitutive heat-shock protein, Hsp-70 (19). In contrast, the ESCRT-independent pathway regulates MVBs assembly and requires Hsp70-phospholipid interactions and the activity of acid sphingomyelinase (nSMase), which can hydrolyze sphingomyelin in the absence of ESCRTs (20). After the formation of MVBs, some of them fuse with lysosomes to degrade cellular components, whereas others fuse with the membrane and are secreted into the extracellular milieu to become exosomes (21). These exosomes then flow with the body fluids, or act in autocrine and paracrine manners to impact recipient cells (22) (**Figure 2**). To date, studies have showed that exosomes can enter the recipient cell cytosol *via* phagocytosis, endocytosis, or micropinocytosis, and subsequently release their cargos to interact with the recipient cell surface in a protein–protein interaction and induce internalization, or activate intracellular signaling cascades without being internalized. In the future, more investigations are needed to comprehensively identify how exosomes can be produced, released and internalized into cells to exert their effects.

## Role of EVs in T2D by Regulating Inflammation

### Inflammation as an Important Factor in T2D Pathogenesis

Inflammation has the physiological purpose to maintain tissue homeostasis. However, uncontrolled inflammation leads to tissue damage and diseases (23). It is well-known that chronic inflammation is a universal and potentially unifying mechanism of metabolic diseases (24–27), and some experts have considered T2D as an inflammatory disease (10). Many systemic inflammatory markers, including white blood cell counts, acute-phase proteins (C-reactive protein), and pro-inflammatory cytokines and chemokines are elevated in patients with obesity and T2D. In contrast, when their expressions reduced because of lifestyle changes, pharmacological drugs or other factors, both IR and β-cell failure were significantly improved (28–32). In addition, some studies also showed that inflammation can mediate IR independently from the degree of obesity (33–35).

As the major immune cells, macrophages have been identified as key determinants of local inflammation and insulin sensitivity during adiposity and T2D. In adipose tissue, the accumulation of activated macrophages promoted the expression of pro-inflammatory cytokines (e.g., TNF-α), which then impaired local insulin signaling (24, 36). When these cytokines were sufficient, they were released into circulation, thereby targeting distant sites and worsening systemic IR. In contrast, when the number of macrophages is reduced, the expression of pro-inflammatory cytokines in both adipose tissue and circulation decreased, alleviating IR. Except for adipose tissue, macrophages activated in other major metabolic tissues, such as liver, muscle, and islet, may exert similar effects to aggravate diabetic damage during T2D (37–39). These studies highlight the important role of inflammation in T2D. Here, we suggest that EVs participate in the induction of inflammation in T2D thanks to their abilities to activate macrophages or carry various pro-inflammatory factors, and that modulating EVs or their cargos may be a promising approach for relieving inflammation in T2D.

### Role of Adipose Tissue-Derived EVs in T2D by Regulating Macrophage Activation

Excessive accumulation of fat in adipose tissue has been considered as a major risk factor for obesity and obesity-related T2D (40, 41). Subsequently, the damaged adipocytes change the expressions of exosomal cargos, resulting in low-grade and chronic inflammation, and ultimately systemic IR.

It has been demonstrated that adipose tissue-derived EVs can modulate inflammatory states in T2D by activating macrophages in recipient cells. In 2009, Deng et al. reported that intravenous injection of retinal binding protein 4 (RBP4)-containing EVs isolated from the adipose tissue of obese mice into lean mice facilitated the differentiation of monocytes into macrophages, which led to increased IL-6 and TNFα secretion followed by the development of IR. And this process requires the TLR4/TRIF pathway (42). Zhang et al. observed that adipocyte-derived EVs from obese mice significantly enhanced M1 macrophage polarization and caused IR in adipocyte, probably due to the upregulation of miR-155 (43). Subsequently, sonic hedgehog (Shh), a protein that can stimulate the secretion of inflammatory cytokines from macrophages, has been detected in EVs derived from 3T3-L1 adipocytes. Injecting these EVs into bone marrow (BM)-derived macrophages significantly mediated M1 polarization of macrophages *via* the Ptch/PI3K signaling pathway, which then led to IR in adipocytes (44). However, the roles of EVs in activating other immune cells, such as neutrophils, to promote inflammation have not been identified, which require further exploration.

### Role of Liver-Derived EVs in T2D by Regulating Macrophage Activation

The liver is densely packed with macrophages, known as Kupffer cells, which can be activated during obesity to mediate inflammation and IR in the liver (38). In a recent study, Hirsova et al. (45) reported that fatty acid palmitate promoted the secretion of hepatocyte EVs in a death receptor 5 (DR5) signaling-dependent manner, which then activated an inflammatory macrophage phenotype and increased the release of pro-inflammatory cytokines. In contrast, suppressing the mediators of the DR5 signaling reversed these effects and decreased liver injury. Since obesity-induced inflammation and hepatocyte dysfunction have been confirmed as essential events during the progress of T2D, these EVs may also play important roles in T2D by activating macrophages.

### Role of EVs in T2D by Targeting Effector Organs or Carrying Pro-Inflammatory Factors

In addition to targeting macrophages, EVs can regulate inflammation by directly acting on effector organs. In this regard, altered expressions of 55 miRNAs have been detected in adipose tissue-derived EVs isolated from patients with obesity, leading to altered TGF-β and Wnt/β-catenin signaling in lung epithelial cells, which are important regulators of obesity-induced inflammation (46). Additionally, human M1 macrophage-derived EVs incubated with adipocytes significantly reduced the abundance of differentiated adipocytes, as well as insulin signal transduction and glucose uptake through NF-κB activation (47). Similarly, macrophages pretreated with high glucose showed increased expression of miR-21-5p in macrophage-derived EVs, which promoted the activation of inflammation and regulated podocyte injury in diabetic nephropathy (DN) mice (48). Notably, EVs can also transport cytokines or other pro-inflammatory mediators to modulate the inflammatory state. Wu et al. observed altered levels of inflammatory proteins in plasma-derived EVs from individuals with diabetes, which were strongly associated with the severity of diabetes. And higher levels of vascular endothelial growth factor A (VEGF-A) in EVs play important roles in T2D-related peripheral vascular disease (49). These findings exactly reveal new mechanisms for activating inflammation in T2D.

## Role of EVs in T2D by Regulating Insulin Signaling

**Figure 3:**
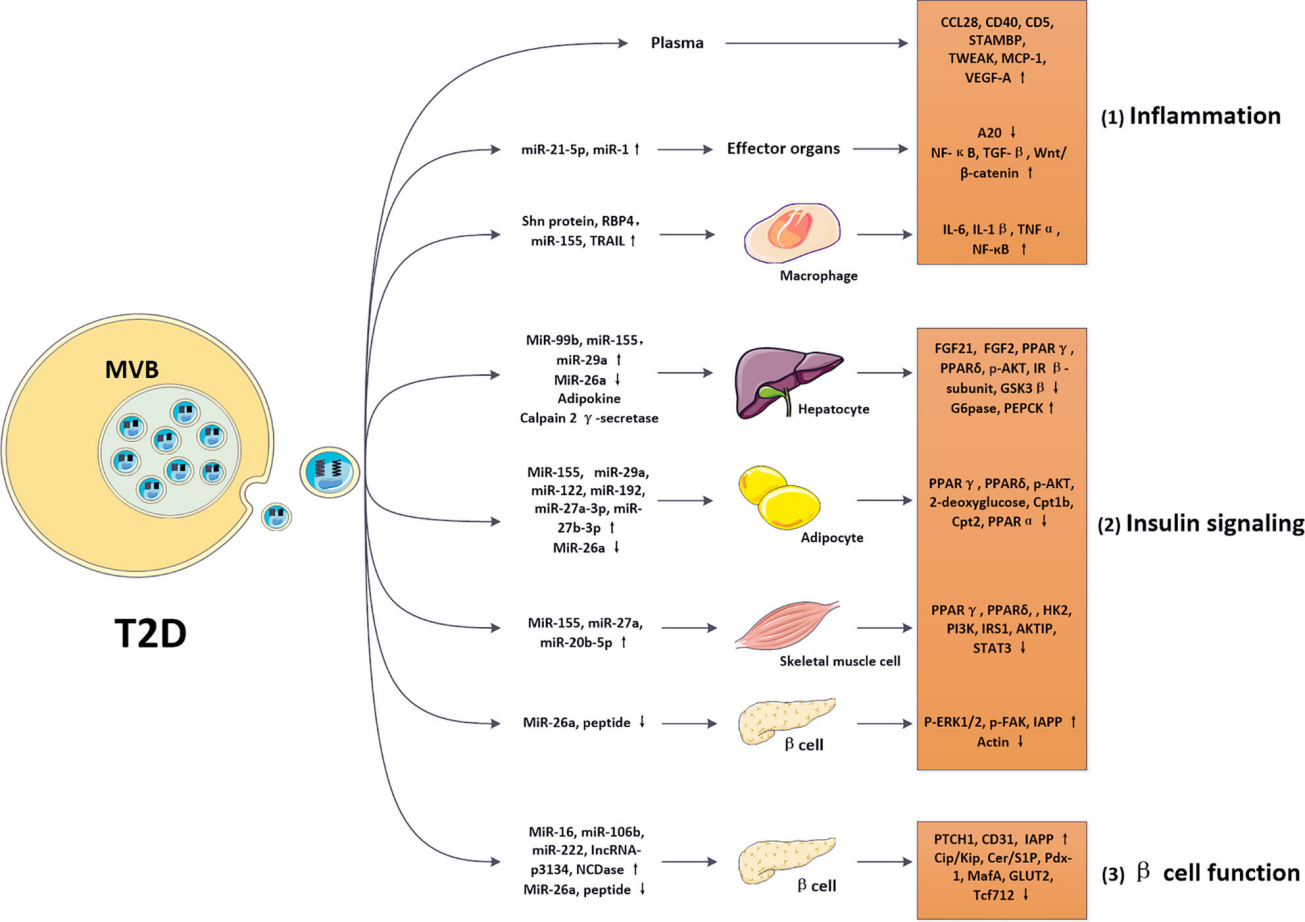
Roles of EVs in regulating diabetic pathological process. In diabetes, altered EVs carrying nucleic acids and proteins can be internalized by various cell types, such as macrophages, adipocytes, hepatocytes, skeletal muscle cells and pancreatic β cells, mediating intercellular communications. 1) Exosomal cargos (such as Shn protein) were modified in the diabetic model and targeted macrophages *via* regulating various cytokines (IL-6, IL-1β, TNFα, and NF-кB). Then, these altered pathways modulated inflammatory responses in T2D. 2) Exosomal cargos (such as miR-99b, miR-155, miR-29a, miR-26a, adipokine, and calpain 2 γ-secretase) were modified in the diabetic model and targeted hepatocytes *via* regulating various cytokines (FGF21, FGF2, PPARγ, PPARδ, p-AKT, IR β-subunit, GSK3β, G6pase, PEPCK). Exosomal cargos (such as miR-155, miR-29a, miR-122, miR-192, miR-27a-3p, miR-27b-3p, and miR-26a) were modified in the diabetic model and targeted adipocytes *via* regulating various cytokines (PPARγ, PPARδ, p-AKT, 2-deoxyglucose, Cpt1b, Cpt2, and PPARα). Exosomal cargos (such as miR-155, miR-29a, miR-29c, miR-27a, miR-23b, miR-27b, and miR-20b-5p) were modified in the diabetic model and targeted skeletal muscle cells *via* regulating various cytokines (PPARγ, PPARδ, PGC-1α, HK2, PI3K, IRS1, AKTIP, STAT3, and p53). Exosomal cargos (such as miR-26a, and peptide) were modified in the diabetic model and targeted β cells *via* regulating various cytokines (p-ERK1/2, p-FAK, IAPP, and actin). These altered pathways modulated insulin signaling in T2D. 3) Exosomal cargos (such as miR-16, miR-106b, miR-222, IncRNA-p3134, NCDase, miR-26a, and peptide) were modified in the diabetic model and targeted β cells *via* regulating various cytokines (PTCH1, CD31, IAPP, Cip/Kip, Cer/S1P, Pdx-1, MafA, GLUT2, and Tcf712). These altered pathways modulated β cell function in T2D. The modifications in inflammation, insulin signaling, and β cell function promoted IR and β cell failure, contributing to the pathology of T2D.

Multiple organs participate in regulating glucose levels in T2D patients, wherein adipose tissue, liver, and muscle have been considered as primary targets of insulin and are vital in the regulation of glucose/fat homeostasis (45, 50–53). After binding of insulin to its receptors on the surface of target sites, numerous insulin signaling pathways can be activated, which promote the synthesis of glycogen and fat and decrease blood glucose levels. Recently, studies showed that abnormal EVs in T2D can alter the activation of insulin signaling in these tissues, which then disrupted normal metabolic responses of recipient sites to insulin and eventually led to IR. Here, we introduce the effects of EVs on metabolic functions and insulin sensitivity of these target organs through disrupting insulin signaling (**Figure 3**).

### Role of Adipose Tissue-Derived EVs in T2D

As a major metabolic site, adipocyte can secrete a variety of EVs, which can be transferred to insulin target cell types, such as hepatocytes, muscle cells and neighboring adipocytes, resulting in impaired insulin signaling and metabolic dysfunction. For example, in a mouse model in which the miRNA-processing enzyme Dicer was specifically knockout in adipocytes, circulating exosomal miRNAs levels were significantly decreased, accompanied with impaired glucose tolerance, whereas transplantation of brown adipose tissue or injection of normal serum EVs restored its expression and improved insulin sensitivity. These effects were partly associated with inhibition of hepatic fibroblast growth factor 21 (FGF21) mRNA and circulating FGF2 expression mediated by serum exosomal transfer of miR-99b from adipose tissue to the liver (54). Besides, lean mice treated with EVs isolated from the adipose tissue macrophages of obese mice also caused glucose intolerance and IR, which was mediated by the upregulation of exosomal miR-155 and downregulation of peroxisome proliferator-activated receptor γ (PPARγ), whereas miR-155 knockout in obese mice reversed these effects (55). Additionally, exosomal miR-29a transported from adipose tissue macrophages to adipocytes, myocytes and hepatocytes reduced the expression of PPARδ and led to IR *in vitro* and *in vivo*, whereas the PPAR δ agonist GW501516 partly reversed miR-29a-mediated IR (56). Furthermore, crosstalk between adipose tissue and skeletal muscle mediated by EVs has also been reported. In patients with obesity-related prediabetes and T2D, circulating exosomal miR‐27a is highly expressed, which is mainly released from adipose tissue and positively associated with IR. Incubation with adipose tissue-derived exosomal miR-27a from obese mice significantly impaired local insulin sensitivity in skeletal muscle C2C12 cells, partly by suppressing the expression of PPARγ and its downstream obesity-related genes (57).

Impaired activation of AKT in response to insulin is a central event in the development of IR and T2D (58), besides, it’s also an important target gene for adipose tissue-derived EVs. An *in vitro* study reported that EVs released from human adipose tissue can stimulate or inhibit insulin-induced AKT phosphorylation in hepatocytes, which may depend on the amount of pro-inflammatory adipokines, thereby modulating systemic IR (59). Similarly, another study using hypoxic adipocytes-derived EVs identified significantly impaired insulin-stimulated 2-deoxyglucose uptake and phosphorylation of AKT in recipient adipocytes (60).

### Role of Liver-Derived EVs in T2D

The liver is another critical endocrine organ regulating glucose metabolism by releasing or storing glucose. Almost all cell types in the liver can release EVs and are targets of systemic EVs derived from other tissues (61). Hepatic EVs-derived miR-130a-3p was showed to attenuate glucose intolerance by inhibiting the PHLPP2 gene in adipocytes (62). Besides, incubation of hepatic stellate cells with EVs derived from hepatocytes undergoing palmitic acid challenge efficiently mediated cell activation and increased the expression of profibrogenic genes, which may be attributed to the alterations in exosomal miRNAs, including miR-128-3p, miR-122, and miR-192 and downregulation of PPARγ (63). Interestingly, recent researches showed that the insulin receptor β-subunit in hepatocytes was sequentially cleaved by calpain 2 and γ-secretase of hepatocyte-derived EVs under hyperglycemic conditions (64), suggesting a novel approach for EVs in regulating T2D by influencing insulin receptors.

### Role of Muscle-Derived EVs in T2D

Skeletal muscle has been showed to secrete various molecules, generally called exerkines to regulate metabolic functions. In T2D conditions, the secretory function of skeletal muscle can be destroyed, which may alter the expression of exerkines, impairing glucose tolerance and causing IR. Recently, increasing studies have proposed that altered EVs are partially responsible for these metabolic dysfunctions (65, 66). Consistent with these findings, a significant increase in the release of EVs from skeletal muscle cells has been reported in high-fat-fed mice. These changes can also alter the expression of genes involved in cell cycle regulation and muscle differentiation *in vitro* (67).

Currently, studies have identified that a sedentary lifestyle can lead to a rapid IR through influencing the expression of genes involved in insulin signaling; and cause systemic mild inflammation, which can be reversed by physical activity (68, 69). Further studies reported that exercise can affect the secretion of muscle-derived EVs, and the contents of circulating EVs increase in an intensity-dependent manner in response to exercise, which suggest potential roles of EVs in the benefits of exercise (70). Moreover, a recent review argued that most multisystemic responses to endurance exercise that relieve the negative effects of obesity and/or T2D are mediated by exerkines released within EVs, and the discovery that 75% of reported myokines and exerkines were present within EVs supports this hypothesis (70).

All these findings suggest that muscle-derived EVs could be involved in the regulation of insulin signaling and metabolic homeostasis in T2D; and that they may also be responsible for the systemic health benefits of exercise.

### Role of β Cell-Derived EVs in T2D

Pancreatic β cells, which secrete insulin, are critical for maintaining glucose homeostasis. Traditionally, β cell secretions were thought to modulate glucose metabolism mainly by regulating insulin level and β cell functions; however, recently, these cells were also showed to play important roles in regulating peripheral insulin sensitivity. For example, the level of miR-26a in β cell-derived EVs was found to be decreased in obese animals, whereas upregulation of miR-26a expression in β cells improved obesity-induced IR in a paracrine manner through circulating EVs (71). Islet amyloid polypeptide (IAPP) is the major component of amyloid deposits found in the islets of patients with T2D. It can be secreted in conjunction with insulin by β cells to regulate glucose metabolism. A recent study discovered that β cell-derived EVs from healthy controls suppressed the aggregation of IAPP by peptide scavenging, whereas T2D β cell-derived and circulating-derived EVs had no such effect (72). These observations provide a new insight for studying the role of β cell-derived EVs in T2D.

### Role of Circulating EVs in T2D

Additionally, some circulating exosomal miRNAs whose secretory sites are unclear have also been discovered to play important roles in T2D. For example, one study showed that EVs isolated from the plasma of obese mice induced glucose intolerance and dyslipidemia in lean mice. These dysfunctions may be caused by increased levels of exosomal miR-122, miR-192, miR-27a-3p, and miR-27b-3p, since incubation with these miRNAs mimicked their effects in lean mice (73). In human studies, circulating EVs derived from obese patients significantly impaired insulin signaling by downregulating the expression of phosphorylated glycogen synthase kinase 3β (GSK3β) and upregulating the mRNA expression of glucose 6-phosphatase (G6pase) and phosphoenolpyruvate carboxy kinase (PEPCK) in HepG2 cells, as well as decreased FGF21 secretion (74). In addition, the circulating level of EV-derived miR-20b-5p has been observed upregulated in T2D. Incubation with circulating miR-20b-5p significantly increased glycogen accumulation and impaired insulin-stimulated glucose metabolism in human skeletal muscle cells, partially by regulating the expressions of AKT-interacting protein (AKTIP) and transporting signal transducer and activator of transcription 3 (STAT3) (75). Other researchers observed that circulating EVs obtained from obese individuals impaired insulin-induced 2-deoxyglucose uptake in 3T3-L1 adipocytes (60). Moreover, modified miRNAs contained in circulating EVs of T2D patients have been considered to participate in regulating the adiponectin pathway (76). As a decrease in plasma adiponectin level can inhibit the expression of adenosine monophosphate kinase (AMPK), which are supposed to improve insulin sensitivity (77), these EVs may also be involved in regulating insulin sensitivity in T2D.

## Role of EVs in T2D by Regulating β Cells Mass

The pancreas is a critical endocrine organ responsible for insulin secretion and maintaining metabolic homeostasis. Emerging studies have indicated that several diabetes-derived EVs can specifically target pancreatic β-cells, thereby modulating β-cell mass. For example, injection of EVs isolated from the skeletal muscle of obese mice specifically targeted the mouse pancreatic cells and induced pancreatic β-cell proliferation in MIN6B1 cells. These effects may be attributed to upregulation of miR-16, which affected the expression of proliferative genes, such as proliferation suppressor protein patched homolog 1 (PTCH1) (78). Additionally, two miRNAs (miR-106b and miR-222) derived from BM cells were found to be increased in islet cells, which may be responsible for BM transplantation-induced β-cell regeneration, and accompanied by downregulation of the Cip/Kip family (79). In islets of streptozotocin (STZ)-induced diabetic mice, EVs isolated from β-cells also improved IR, increased insulin secretion, and preserved the architecture and enhanced the angiogenesis of islets (80). Furthermore, experiments using insulin-secreting INS-1 cells found that the secretion of neutral ceramidase (NCDase) *via* EVs was increased, whereas treatment with EV-packaged-NCDase derived from INS-1 cells effectively inhibited palmitate-induced β-cells apoptosis by regulating the sphingolipid-induced signaling pathway (81). Previous studies have demonstrated that the formation process of IAPP may promote β-cell dysfunction in T2D patients (82). Since β cell-derived EVs from healthy controls suppressed the aggregation of IAPP by peptide scavenging, whereas T2D β cell-derived and circulating-derived EVs have no effect, the aggregation of IAPP in T2D may lead to β cell failure (72). Long noncoding RNAs (lncRNAs) secreted by EVs can also exert critical effects. In T2D patients, the circulating level of exosomal lncRNA-p3134 were found to be increased; this lncRNA is associated with fasting blood glucose and homeostasis model assessment β-cell function (HOMA-β) levels, whereas its level in β-cells was downregulated. As lncRNA-p3134 can help preserve β-cell function and positively regulate glucose-stimulated insulin secretion (GSIS) by upregulating key factors (Pdx-1, MafA, GLUT2, and Tcf7l2) in β cells, these results provide a new mechanism for β cell regulation *via* lncRNAs (83) (**Figure 3**).

## Clinical Therapeutic Potential of EVs

With the increased understandings of the crucial roles of EVs in pathogenesis of T2D, their potentials for clinical use in T2D patients as diagnostic and therapeutic tools have attracted increased attentions. Here, we summarize the current advances in the potential applications of EVs in biomarker discovery, intervention monitoring and machinery discovery, therapeutic strategy discovery and drug delivery in T2D and its complications.

### Biomarker Discovery

Total circulating molecules have great potential to serve as T2D biomarkers since they are easily detected in body fluids. In this regard, several serum/plasma miRNAs levels have been identified to be strongly associated with T2D pathology (84–89), and even associated with prediabetes (84, 85, 90). In recent years, characterizing EVs containing various cellular molecules from human biofluids has also gained increased attentions, thanks to their ability to resist enzymatic degradation through a lipid bilayer protection (75, 76, 91, 92). So far, numerous EVs circulating in the body fluids have been found altered in T2D, which are closely related to immunity and metabolic dysfunctions, while some were found altered in T2D complications. Here, we briefly summarize the molecules currently discovered to be altered in T2D and its complications (**Table 1**). Further in-depth studies aimed at characterizing EVs and their functional cargos are needed to promote the discovery of novel biomarkers, thereby assisting in T2D diagnosis and timely implementation of personalized therapies.

**Table 1:** Molecules in cirulating EVs as biomarkers of T2D.

Cargo	Expression	Circulating source	Compared subject	Effect	Reference
miR-375-3p	Increased	Serum	STZ-injected mice vs. vehicle-injected mice newly diagnosed T2D patients vs. NGT subjects	Exosomal miR-375-3p increased in circulation of STZ-injected mouse prior to hyperglycemia and in new-onset T2D patients, serving as a potential biomarker of islets damage	Fu et al. (91)
miR-20b-5p	Increased	Serum	T2D patients vs. subjects with impaired glucose tolerance vs. NGT subjects	MiR-20b-5p overexpression reduced expression of AKTIP, STAT3 and insulin-stimulated glycogen accumulation in human skeletal muscle cells	Katayama et al. (75)
miR-320a miR-197, miR-509-5p	Increased Decreased	Serum Plasma	Metabolic syndrome patients vs. T2D patients vs. hypercholesterolemia patients vs. hypertension patients vs. healthy controls	Differentially expressed exosomal miRNAs are involved in T2D related pathways, and they could also be dysregulated in collective metabolic disorders	Karolina et al. (92)
Leptin receptor, p-insulin receptor, p-S6RP, p-GSK3β, p-AKT FGF21	Decreased Increased	Plasma	T2D patients vs. euglycemic subjects T2D patients vs. obesity-matched euglycemic subjects	Exosomal leptin receptor and p-insulin receptor levels are negatively correlated with the risk of developing T2D. Exosomal p-AKT, phospho-GSK3β and phospho-S6RP levels are negatively associated with HOMA-B or HOMA-IR	Freeman et al. (8)
miR-326 let-7a, let-7f	Increased Decreased	Plasma	Treatment-naïve and poorly controlled T2D patients vs. nondiabetic subjects	Poorly controlled T2D is associated with the aberrant levels of let-7a/let-7f and miR-326 in circulating EVs. Circulating exosomal miR-326 levels are inversely correlated with its putative target adiponectin	Santovito et al. (76)
miR-133b, miR-342, miR-30a	Increased	Urine	T2D patient with microalbuminuria and macroalbuminuria vs. T2D patient with normoalbuminuria vs. healthy control	These urinary exosomal miRNAs could be potential biomarkers for T2DN and have a synergistic effect in T2DN pathogenesis, though they could also altered in some normoalbuminuria cases	Eissa et al. (93)
let-7c-5p miR-29c-5p, miR-15b-5p	Increased Decreased	Urine	T2D patient with DN vs. T2D patient without DN vs. healthy controls	Urinary exosomal let-7c-5p is correlated with both renal function and progression of T2DN	Li et al. (94)
miR-362-3p, miR-877-3p, miR-150-5p, miR-15a-5p	Increased Decreased	Urine	T2D patient with no renal disease vs. T2D patient with macroalbuminuria	These urinary miRNAs might be novel biomarkers for incipient diabetic kidney disease, and might regulate DN through p53, mTOR, and AMPK pathways	Xie et al. (95)
Aquaporin 5 and 2	Increased	Urine	T2D patient with DN vs. T2D patient with proteinuric nondiabetic nephropathy vs. T2D patient with normal renal function	Aquaporin 5 and 2 could be potential biomarkers to help in classifying the clinical stage of DN and positively correlated with the progression of the DN	Rossi et al. (96)
miR-320c, miR-6068	Increased	Urine	T2D patient with DN vs. T2D patient without DN vs. healthy controls	Deregulated miR-320c might be indirectly involved in TGF-β signaling *via* targeting TSP-1 and may represent a novel candidate marker for early progression of T2DN	Delić et al. (97)
miR-15b, miR-34a, miR-636	Increased	Urine	T2D patient with albuminuria vs. T2D patient with normoalbuminuria vs. healthy control	These urinary exosomal miRNAs might be novel biomarkers for early diagnosis of T2DN. A significant correlation is existed between the three selected miRNAs	Eissa et al. (98)

T2D, type 2 diabetes,; EVs, extracellular vesicles; IR, insulin resistance; NGT, normal glucose tolerance; T2DN, T2D nephropathy.

### Interventions Monitoring and Machinery Discovery

Antidiabetic drugs currently in use mainly include thiazolidinediones (TZDs), metformin, sulfonylureas, and sodium-glucose cotransporter 2 (SGLT2) inhibitors. Recently, studies suggested that some metabolic drugs can influence the generation, release, and composition of EVs in T2D patients, indicating EVs as a possible platform for intervention monitoring. In this regard, metformin has been found to suppress cleavage of the insulin receptor and inhibit calpain 2 release in EVs, thus, re-establishing insulin signaling and enhancing insulin sensitivity (64). It can also decrease the levels of multiple T2D-affected miRNAs in EVs even close to those in healthy controls, which may help monitor the metformin response in T2D patients (99). In a randomized controlled trial, the levels of circulating endothelial microparticles and endothelial progenitor cells, as well as their ratio showed greater changes following treatment with pioglitazone treatment versus metformin, representing a better endothelial repair capacity in newly diagnosed T2D patients (100). However, it remains unclear whether these alterations resulted from modulation of the related pathophysiology or were caused by improved glucose levels.

In addition to drug treatments, other important approaches to T2D treatment, such as dietary changes, exercise and bariatric surgery, can also modulate EV levels and subtypes. For example, intervention with an oat-enriched diet in T2D subjects reduced fibrinogen- and tissue factor-related platelet microparticles and CD11b-positive monocyte microparticles, which can serve as markers of metabolic health, and assess the effects of a well-controlled diet in T2D (101). Exercise can reverse T2D inflammation and IR related to T2D and triggers rapid release of EVs into the circulation, which may participate in intercellular communication and act as important mediators of adaptation processes to exercise (102). The beneficial effects of bariatric surgery *via* regulating EVs have also been explored. Transient alterations in circulating EV- and plasma-derived fatty acid binding protein 4 (FABP4) has been detected after bariatric surgery, reflecting changes in adipose tissue homeostasis (103). It also reduced CD36-bearing EVs of endothelial and monocyte origin, suggesting improvements in ectopic fat deposition, oxidative stress, and low-grade inflammation (104). Additionally, weight loss following gastric bypass surgery led to modification of the circulating adipocyte-derived exosomal miRNA profile, correlating with improvements in both IR and glucose homeostasis (105). To date, since more and more EVs have been identified to be influenced by therapeutic intervention, a deeper understanding of EV biogenesis and their functional cargos might open new prospects for monitoring the efficacy of therapeutic interventions and favor machinery discovery.

### Therapeutic Agents Discovery

Though a number of EVs have been identified to participate in the pathogenesis of T2D, actually, there are also some EVs possessed beneficial effects and can served as therapeutic agents for T2D and its complications. For instance, miR-26a is reduced in serum EVs from patients with obesity and is inversely correlated with the clinical features of T2D. Using miR-26a knockin and knockout mouse models, researchers found that overexpression of miR-26a in β cells significantly enhanced peripheral insulin sensitivity in a paracrine manner through circulating EVs (71). Additionally, EVs derived from mouse brain endothelial cells promoted neurorestorative effects after stroke in T2D mice, which were mediated by upregulation of miR-126 (106). In addition, overexpression of heat shock protein 20 (Hsp20) in Hsp20-transgenic cardiomyocytes improved cardiac function and angiogenesis in diabetic hearts by releasing instrumental EVs. Hsp20-engineered EVs may be a new therapeutic approach for diabetic cardiomyopathy (107). More studies on the therapeutic effects of EVs in T2D and its complications are listed in **Table 2**.

**Table 2:** The application of EVs and mimics in the therapy of T2D and its complications.

Active molecule	Source	Disease	Effect	Reference
**Therapeutic agents**				
MiR-26a	β cells	T2D	MiR-26a in β cells significantly enhanced peripheral insulin sensitivity in a paracrine manner through circulating EVs	Xu et al. (71)
MiR-126 and miR-296	Endothelial progenitor cells	T2D	EVs released from endothelial progenitor cells activated the PI3K-AKT and eNOS signaling pathways, promoted insulin secretion, survival as well as induced islet angiogenesis	Cantaluppi et al. (108)
MiR-106b and miR-222	BM cells	T2D	MiR-106b and miR-222 were secreted by BM cells and increased in islet cells after BMT, inducing β-cell regeneration, probably through Cip/Kip family down-regulation	Tsukita et al. (79)
MiR-16	Skeletal muscle cells	T2D	EVs isolated from obese mice targeted pancreas and induced β-cell proliferation *via* the upregulations of miR-16, which affected the expression of proliferative genes, such as PTCH1	Jalabert et al. (78)
Unrevealed	β cells	T2D	EVs derived from β-cells play a role in preserving pancreatic islet architecture and its function, and in inducing islet angiogenesis	Sun et al. (80)
NCDase	INS-1 cells	T2D	Treatment with EV-packaged-NCDase derived from INS-1 cells effectively inhibited β-cells apoptosis through regulating the sphingolipid-induced signaling pathway	Tang et al. (81)
MiR-126	Brain endothelial cells	Stroke	EVs derived from brain endothelial cells promoted neurorestorative effects after stroke	Venkat et al. (106)
Hsp20	Cardiomyocytes	DCM	Overexpression of Hsp20 in cardiomyocytes improved cardiac function and angiogenesis *via* the release of instrumental EVs	Wang et al. (107)
MiR-146a	Brain endothelial cells	Cognitive impairment	The delivery of brain endothelial cell-derived EVs loaded with miR-146a downregulated PrP(c) levels and restored short term memory function	Kalani et al. (109)
bFGF, PDGFBB, and TGF-β	PRPs	Diabetic wound	PRP-derived EVs induced proliferation and migration of endothelial cells and fibroblasts to improve angiogenesis and re-epithelialization in chronic wounds	Guo et al. (110)
**MSC-derived EV-based therapy**				
Unrevealed	ADSCs	Obesity	ADSC-derived EVs alternatively polarized M2 macrophages, reduced inflammation and promoted white adipose tissue beiging by STAT3 to the macrophages, and finally improved insulin sensitivity	Zhao et al. (111)
Unrevealed	MSCs	T2D	MSC-derived EVs improved hepatic glucose and lipid metabolism by activating autophagy *via* the AMPK pathway	He et al. (112)
Unrevealed	MSCs	T2D	MSC-derived EVs reversed IR and increase β-cell survival, accompanied with the increased phosphorylation of IRS-1 and AKT and the increased expression of GLUT4 in muscle	Sun et al. (113)
Unrevealed	MSCs	DN	MSC-derived EVs exerted an anti-apoptotic effect and protected tight junction structure in tubular epithelial cells	Nagaishi et al. (114)
Unrevealed	MSCs and HLSCs	DN	Stem cell-derived EVs inhibited fibrosis and prevented the progression of diabetes-induced chronic kidney injury	Grange et al. (115)
Unrevealed	MSCs	DN	MSC-derived EVs improved renal function and showed histological restoration of renal tissues *via* inducing autophagy	Ebrahim et al. (116)
MiR-486	ADSCs	DN	ADSCs-derived EVs relieved DN symptom by promoting autophagy flux and reducing podocyte apoptosis *via* regulating miR-486/Smad1/mTOR signaling pathway	Jin et al. (117)
MiR-26a-5p	ADSCs	DN	ADSC-derived EVs transferred miR-26a-5p to glomerular podocytes, which ameliorated DN pathology	Duan et al. (118)
MiR-126, miR-130a, miR-132, miR-let7b, and miR-let7c	ADSCs	Erectile dysfunction	ADSC-derived EVs induced the proliferation of endothelial cells and restored erectile function *in vivo*, as well as decreased fibrosis of corpus cavernosum	Zhu et al. (119)
Unrevealed	ADSCs	Erectile dysfunction	ADSC-derived EVs rescued corpus cavernosum endothelial and smooth muscle cells by inhibiting apoptosis and promoted the recovery of erectile function	Chen et al. (120)
MiR-146a	MSCs	Cognitive impairment	Exosomal miR-146a secreted by MSCs exerted anti-inflammatory effects on damaged astrocytes and prevented diabetes-induced cognitive impairment	Kubota et al. (121)
Unrevealed	MSCs	Cognitive impairment	MSC-derived EVs recovered diabetes-induced cognition impairment and histologic abnormity	Zhao et al. (122)
Unrevealed	MSCs	Cognitive impairment	MSC-derived MVs improved cognitive impairment and histological abnormalities	Nakano et al. (123)
Unrevealed	MenSCs	Diabetic wound	MenSCs-derived EVs accelerated re-epithelialization through activating the NF-κB signaling way, thereby promoting cutaneous healing process	Dalirfardouei et al. (124)
MiR29c and miR150	nAT-MSCs	Diabetic wound	MSC-derived MVs improved their migration ability and wound healing ability by altering the expression of genes associated with cell migration, survival, inflammation, and angiogenesis as well as miR29c and miR150	Trinh et al. (125)
lncRNA H19	MSCs	Diabetic wound	MSC-derived exosomal lncRNA H19 prevented the apoptosis and inflammation of fibroblasts by impairing miR-152-3p-mediated PTEN inhibition, leading to the stimulated wound-healing process	Li et al. (126)
DMBT1 protein	USCs	Diabetic wound	USC-derived EVs treated diabetic soft tissue wound healing by promoting angiogenesis *via* transferring DMBT1 protein	Chen et al. (127)
Unrevealed	MSCs	DPN	MSC-derived EVs attenuated neurovascular dysfunction and promote functional recovery in mice with DPN through inhibiting proinflammatory cytokines	Fan et al. (128)
MiR-126	MSCs	Retinal inflammation	MSC-derived EVs alleviated hyperglycemia-induced retinal inflammation by transferring miR-126 and suppressing the HMGB1 signaling	Zhang et al. (129)
Unrevealed	MSCs	Myocardial injury	MSC-derived EVs improved diabetes-induced myocardial injury and fibrosis *via* inhibiting TGF-β1/Smad2 pathway	Lin et al. (130)
**EV mimics-based delivery system**				
Unrevealed	ESC-derived EV-mimetic nanovesicles	Erectile dysfunction	ESC-derived EV-mimetic nanovesicles completely restored erectile function by promoting penile angiogenesis and neural regeneration	Kwon et al. (131)
miR-21-5p	Engineered ADSCs	Diabetic wound	Engineered human ADSC-derived EVs loaded with miR-21-5p promoted diabetic wound healing through increasing re-epithelialization, collagen remodeling, angiogenesis, and vessel maturation	Lv et al. (132)
lncRNA-H19	EV-mimetic nanovesicles	Diabetic wound	EV-mimetic nanovesicles with a high content of lncRNA-H19 neutralized the suppression of regeneration of hyperglycemia as well as accelerated the chronic wounds healing	Tao et al. (133)

T2D, type 2 diabetes; EV, extracellular vesicles; Hsp20, heat shock protein 20; DCM, diabetic cardiomyopathy; DN, diabetic nephropathy; IRS-1, insulin receptor substrate 1; MSC, mesenchymal stem cell; ADSCs, adipose-derived stem cells; DPN, diabetic peripheral neuropathy; HMGB1, high-mobility group box 1; MenSC, menstrual blood-derived mesenchymal stem cell; nAT-MSCs, non-diabetic adipose tissue-MSCs; USCs, urine-derived stem cells; HLSCs, human liver stem-like cells; PRPs, platelet-rich plasma; ESC, embryonic stem cell; PTCH1, proliferation suppressor protein patched homolog 1; BMT, bone marrow transplantation; NCDase, neutral ceramidase; DMBT1, deleted in malignant brain tumors 1.

### Therapies Based on Mesenchymal Stem/Stromal Cell-Derived EV

MSC therapy is a novel therapeutic strategy emerging in recent years. It can differentiate into diverse cell types and produce a variety of molecules, including EVs (134). Recently, studies found that MSC-derived EVs successfully mimicked the therapeutic effects of MSCs, providing an alternate to MSC transplantation. Compared to the transplantation of live cells, cell-free therapy has many advantages (135), such as lost cost related to storage and maintenance, greater safety, better assessment and control of drug dosage and potency, economical mass-production in specific cell lines due to their stability and modifiability. It also avoids immune compatibility, tumorigenicity, embolism formation, and transmission of infections after MSC transplantation. Moreover, small vesicles easily circulate through the thin capillaries and pass through the blood-brain barrier. Nowadays, MSC-derived EV-based therapy is considered as a promising therapeutic tool for various diseases, including liver injury, myocardial infarction, drug addiction, immunoregulation and cancer (136), and recent studies also suggested that these EVs can play important roles in the treatment of T2D and its complications (**Table 2**). In this regard, human umbilical cord MSC-derived EVs were found to improve hepatic glucose and lipid metabolism in T2D by enhancing autophagy in an AMPK-dependent manner (112). They can also reverse IR and increase β-cell survival in high-fat diet-fed or STZ-induced T2D rats, accompanied with the increased phosphorylation of the insulin receptor substrate 1 (IRS-1) and AKT, as well as increased expression of GLUT4 in the muscle (113). Furthermore, directly injecting MSC-derived EVs into the blood of diabetic mice exerted protective effects by inhibiting β-cell apoptosis (113). Adipose-derived stem cells (ADSCs) are key regulators of obesity-induced inflammation. A recent study showed that EVs derived from ADSCs significantly polarized M2 macrophages, reduced inflammation, and promoted white adipose tissue beiging, at least partially through the effects of STAT3 on the macrophages, which eventually improved insulin sensitivity (111). Furthermore, EVs released from the endothelial progenitor cells activated the PI3K-AKT and eNOS signaling pathways, promoted insulin secretion and cell survival; and induced islet endothelial cell proliferation, differentiation, and angiogenesis; which are associated with packaged proangiogenic miR-126 and miR-296 (108).

MSC-derived EVs can also mediate the protection from diabetic complications. For example, in a mouse model of diabetes, both human ADSCs and their secreted EVs reversed neuropathic pain, maintained the pro/anti-inflammatory cytokine balance, and inhibited skin innervation loss, revealing a promising approach for treating diabetic neuropathic pain (137). Besides, ADSC-derived EVs relieved DN symptoms by promoting the expression of miR-486, which inhibited the Smad1/mTOR signaling pathway in podocytes, increased autophagy flux, and reduced podocyte apoptosis (117). Diabetic peripheral neuropathy (DPN) is an important complication of diabetes. Treatment of DPN with MSC-derived EVs successfully attenuated neurovascular dysfunction and promoted functional recovery in diabetic mice through inhibiting proinflammatory cytokines (128). Moreover, in a diabetes-induced cognitive disorder mouse model, EVs derived from BM-MSCs recovered cognition impairment and histologic abnormities (121, 122).

Regenerative medicine refers to the application of different approaches to promote the regeneration process of lost or damaged tissues, so as to completely replace damaged tissues. In the past few years, emerging studies have indicated that MSC-derived EVs are promising tools for regeneration and repairment of damaged cells, particularly given their high biocompatibility restrictions and cost-effectiveness. To support this idea, EVs released from menstrual blood-derived MSCs were showed to accelerate re-epithelialization, possibly through activating the NF-κB signaling pathway, thereby promoting the cutaneous healing process in diabetic mice (124). Besides, transfection of T2D ADSCs with EVs derived from non-diabetic ADSCs also enhanced their mobility *in vitro* and promoted wound healing *in vivo* (125). In diabetic foot ulcer, MSC-derived exosomal lncRNA H19 promoted fibroblast proliferation and migration, as well as prevented apoptosis and inflammation by upregulating miR-152-3p-mediated PTEN (126). These studies reveal attractive roles of MSC-derived EVs in tissue regeneration and provide a promising method for regenerative medicine.

Despite the encouraging results of the studies on MSC-derived EVs, most studies were preclinical. Thus, additional research is needed to optimize MSC-derived EV-based therapies. First, it is critical to control the source of MSCs and their optimal culture conditions to produce desired cargos in large amounts. Second, the methods used to isolate and purify the desired EVs from MSCs must be improved, since a large number of EVs could be released by MSCs. Third, investigations of the molecular bases underlying different EVs are important, which contribute to the future specific design of artificial EVs. Finally, how to control the dosage of EVs secreted by MSCs to maximize their therapeutic effects and detect their contents in target tissues also require more basic and clinical practice.

### Drug Delivery

Gene therapy is an emerging therapeutic strategy for treating numerous diseases by modulating the expression of target genes. However, the absence of suitable delivery systems greatly hinders its clinical applications. The abilities of EVs to transfer various bioactive molecules to nearby or distant recipient cells provide a novel approach for drug delivery across different organs and target specific sites. Compared to other nucleic acid drug carriers, EVs not only deliver nucleic acid or proteins in an active form, but also prevent immunological damage (138, 139). Besides, EVs contain a double-layer membrane structure that protects their contents from RNases, as well as a supposed recognition system that favors targeting of recipient cells, thereby improving the efficiency and precision of transportation (140). Nowadays, EVs containing desired cargos or drugs have been proposed as promising tools for T2D treatment. Except for natural EVs secreted by various tissues or MSCs, several strategies have been exploited to generate ideal EVs, such as genetically or chemically modifying genes of donor cells to alter EV cargos, or directly loading exogenous nucleic acids or proteins into purified EVs. For example, engineered human ADSC-derived EVs loaded with miR-21-5p mimics by electroporation significantly promoted diabetic wound healing through increasing re-epithelialization, collagen remodeling, angiogenesis, and vessel maturation *in vivo* (132). In addition, due to their high tolerance to the body’s endogenous system, EVs are considered promising natural carriers for small interfering RNA (siRNA) delivery, which has emerged as a therapeutic candidate for gene therapy, without a visible immune response (139).

Given the considerable complexity of exosomal components, wherein most are unidentified and may cause unexpected effects, as well as the related high risk of off-target effects, these events significantly hinder the clinical application of EVs (141). Recently, artificially synthesized EV-mimics, such as liposomes (141) and EV-mimetic nanovesicles (142) have been developed to contain only crucial components of natural EVs, thereby limiting the negative effects of unwanted cargos and enhancing the therapeutic efficiency of the delivered drugs. *In vitro*, studies showed that using EV-mimetic nanovesicles with a high content of lncRNA-H19 as a delivery vehicle neutralized the suppression of regeneration of hyperglycemia and accelerated chronic wound healing (133). In diabetic mice, embryonic stem cell-derived EV-mimetic nanovesicles completely restored the erectile function by promoting penile angiogenesis and neural regeneration, while embryonic stem cell only partly restored erectile function (131). In addition, Sato et al. developed hybrid EVs by fusing their membranes containing specific membrane proteins with liposomes using the freeze-thaw method. This membrane-engineering approach facilitated cellular uptake of the modified EVs and also reduced their circulation time in the blood, enabling the development of an advanced drug delivery system (143). Recent advances in the application of EVs and their mimics in the therapy of T2D and its complications are showed in **Table 2**.

## Conclusions and Prospects

In this review, we summarized the important roles of EVs in the pathogenesis of T2D through regulating inflammation, influencing insulin signaling or directly modulating β cell mass. Besides, they could also serve as attractive diagnostic and therapeutic tools for T2D and its complications. However, there are still a lot of challenges before the clinical use of EVs (144). First, clinical applications of EVs serving as biomarkers or therapeutic agents require high-purity EVs, which are difficult to obtain using the current isolation techniques. Current separation methods have many limitations: inability to prepare a large number of high-purity EVs from biological fluids due to lipoprotein contamination, inability to distinguish between the subtypes of EVs, inability to isolate EVs containing only the expected cargos, and inability to separate EVs targeting specific cells. Besides, it remains unclear how EV biogenesis pathways are implicated in T2D individuals, which then influence the release of EVs and their contents. Third, exosomal cargos and their expressions are not always consistent with their expressions in donor cells, and how these cargos are selected from donor cells is unclear, which increase the difficulty of controlling the type and dosage of components and therapeutic effects of EVs. Moreover, the type of EVs and their cargos are still largely unidentified, as well as their molecule bases in promoting or reversing the T2D pathogenesis. Further investigations aimed at identifying functional molecules in EVs and their underlying mechanisms to influence target cells will be of high impact for the development of EV-based therapeutics. Finally, although altered EVs have been identified as important factors promoting the pathogenesis of T2D, whether their changes are consequences of T2D inflammation, IR, and β cell dysfunction, or these effects are caused by EVs changes remains unclear. In the future, additional efforts are needed to characterize EVs to promote their clinical applications in T2D.

## Author Contributions

JL and XS wrote the manuscript. X-LY, SH, F-LZ, and HJ prepared the figures. Z-NG and YY reviewed and edited the manuscript. All authors contributed to the article and approved the submitted version.

## Funding

This work was supported by the National Natural Science Foundation of China (81971105) to Z-NG, the Program for JLU Science and Technology Innovative Research Team (2017TD-12) and Jilin Provincial Key Laboratory (20190901005JC) to YY.

## Conflict of Interest

The authors declare that the research was conducted in the absence of any commercial or financial relationships that could be construed as a potential conflict of interest.
